# Supplementary material for: Nucleotide-Binding Leucine-Rich Repeat Genes CsRSF1 and CsRSF2 Are Positive Modulators in the Cucumis sativus Defense Response to Sphaerotheca fuliginea
Source: Int J Mol Sci. 2021 Apr 13;22(8):3986. doi: 10.3390/ijms22083986 (PMC8069588; doi:10.3390/ijms22083986)
Supplement: Supplementary file 1 [file ijms-22-03986-s001.pdf]

Supplementary Material

Supplementary Figures and Tables

Supplementary Figures

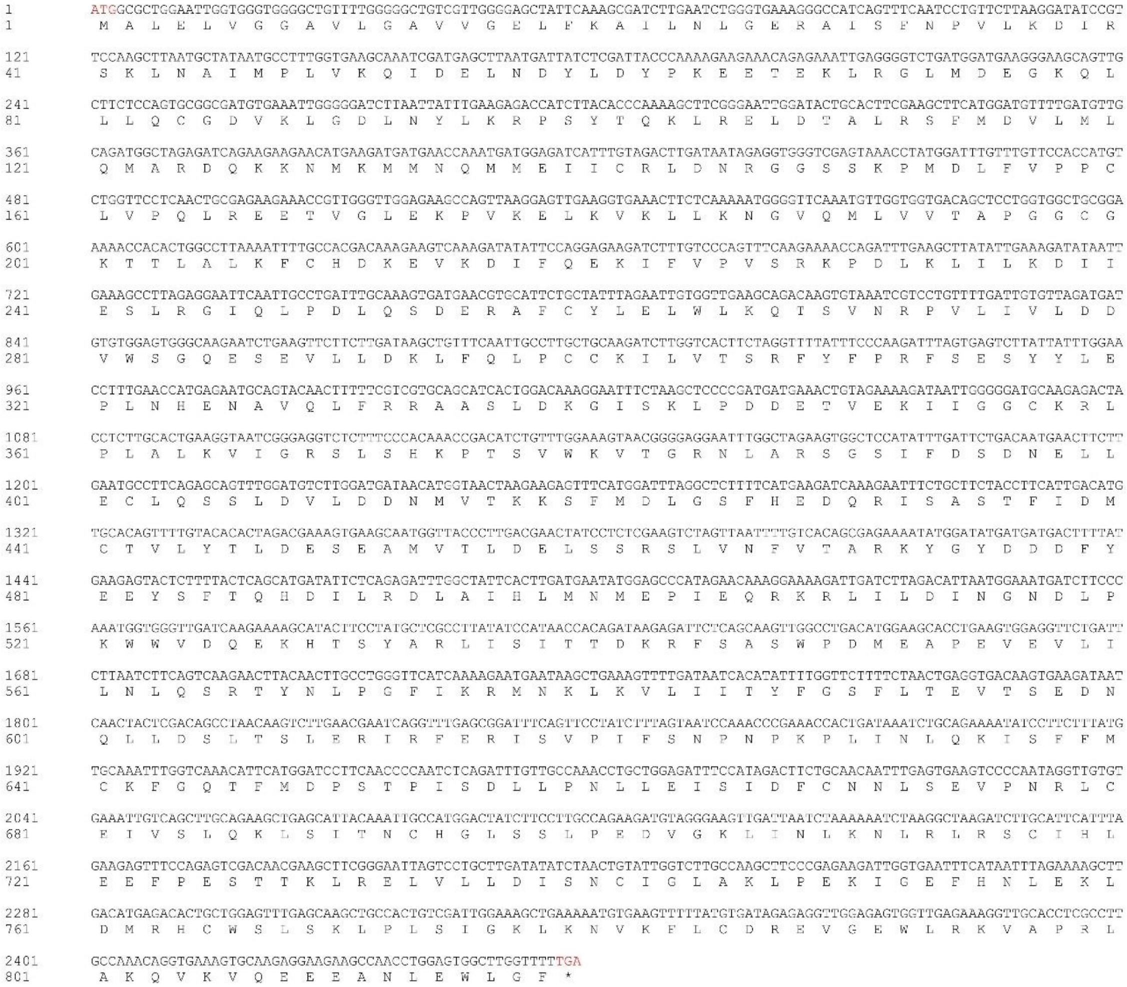

Figure S1. Nucleotide and deduced amino acid sequences of cucumber *CsRSF1* CDS encoding a protein. The deduced amino acid sequences are given below the nucleotide sequences. The transcriptional start site is shown in red and the termination codon is marked by an asterisk (*).

```
1       ATGGCGGTTACAGATTTCTTTGTTGGAGAGATAGCCACTGAGCTTCTCAGAATGATGGTACAACTTTCGACCAAATCCTGCCTTTGTAAAACGACGGCAGCTCAAATCGCCAATTCTATT
1        M  A  V  T  D  F  F  V  G  E  I  A  T  E  L  L  R  M  M  V  Q  L  S  T  K  S  C  L  C  K  T  T  A  A  Q  I  A  N  S  I

121     CAACAAATTCTGCCGATTATTGAAGAGATCAAGTACTCGGGAGTTGAATTACCCGCTCATCGCCAATTTCAGTTAGATCGCTTCAGCGAAACTCTTAGAAGAGGCATCGAGATTTCCGAG
41       Q  Q  I  L  P  I  I  E  E  I  K  Y  S  G  V  E  L  P  A  H  R  Q  F  Q  L  D  R  F  S  E  T  L  R  R  G  I  E  I  S  E

241     AAGGCTCTTCAATGTGGCCGATTAAACATTTACAGAAACTTACGGCTCGCGAGGAAGATGGAGAAGCTTGAAAAGGATATATGTCGATTCATTAATGGCACCATGCAGGCGCATATACTG
81       K  A  L  Q  C  G  R  L  N  I  Y  R  N  L  R  L  A  R  K  M  E  K  L  E  K  D  I  C  R  F  I  N  G  T  M  Q  A  H  I  L

361     GCCGACGTGCATCATATGAGATTCCAGACCACCGAGCGGTTTGACCGGCTTGAAGGTGTTTTGTTGGAGCGGCGGCTTGAGTCGATGAAGATTAGAGCAGATGCTTCGGGAGAGGAAAGG
121      A  D  V  H  H  M  R  F  Q  T  T  E  R  F  D  R  L  E  G  V  L  L  E  R  R  L  E  S  M  K  I  R  A  D  A  S  G  E  E  R

481     TGGTGGGTTGAGGAGGCGTTTAAGAAGGCCGAGGAGGAGGAAAGGTATGAGAGTAATTTCGTGAATATAGGAACTGGATTGCGTGTGGGGAAGAGAAAATTGAAGGAGCTGGTGATTGGA
161      W  W  V  E  E  A  F  K  K  A  E  E  E  E  R  Y  E  S  N  F  V  N  I  G  T  G  L  R  V  G  K  R  K  L  K  E  L  V  I  G

601     AAGGAGGATTTAACGGCGGTTGGGATTAGTGGAATTGGGGGTTCGGGGAAGACTACTTTAGCTAGAGAATTCTGCAAAGATCCGGAAGTTCGAAGACACTTTAAAGAGAGAATTTTGTTC
201      K  E  D  L  T  A  V  G  I  S  G  I  G  G  S  G  K  T  T  L  A  R  E  F  C  K  D  P  E  V  R  R  H  F  K  E  R  I  L  F

721     TTAACGGTGTCACAGTCCCCTGATGTGGAGCAGCTGAGGAGAACGATCTGGGAATTTGTGATGGGTAGTGATAGTGTCAATTCTAATAATTTGATTTTACATGGGAGGCCTTCAAATTCA
241      L  T  V  S  Q  S  P  D  V  E  Q  L  R  R  T  I  W  E  F  V  M  G  S  D  S  V  N  S  N  N  L  I  L  H  G  R  P  S  N  S

841     GCGCTTTTGGTTCTGGATGATGTGTGGTCAATTTCAGTTCTTGAAAATGTTATTCCAAACGTAACTGGTTGCAAAACTCTTGTTGTTTCACGATTCAAATTCCCTGAAGTTCTTAGAGAA
281      A  L  L  V  L  D  D  V  W  S  I  S  V  L  E  N  V  I  P  N  V  T  G  C  K  T  L  V  V  S  R  F  K  F  P  E  V  L  R  E

961     ACTTATGAAGTAGAGTTGTTGAAAGAAAGTGAAGCAATTGCTCTGTTTTGCCACTCAGCTTTCGGACAACAGTCGATTCCTTTGTCTGCTAATCACAACTTGGTCAAACAGGTTGTGAAT
321      T  Y  E  V  E  L  L  K  E  S  E  A  I  A  L  F  C  H  S  A  F  G  Q  Q  S  I  P  L  S  A  N  H  N  L  V  K  Q  V  V  N

1081    GAATGCAAATGTTTGCCTCTGGCTCTTAAAGTCATAGGAGCATCACTCAGAGGACAGAGCGAGATGTTCTGGAATAATGCCAAGTCTAGGTTGTCACGTGGCGAGCCTATTTGCGAGTCC
361      E  C  K  C  L  P  L  A  L  K  V  I  G  A  S  L  R  G  Q  S  E  M  F  W  N  N  A  K  S  R  L  S  R  G  E  P  I  C  E  S

1201    CATGAGAACAAATTGCTTCAAAGAATGGCAATCAGTATTGAACGCCTCTCGAGTAAAGTGAGAGAATGTTTCCTCGACCTGGGATGCTTTCCTGAAGACAAAAGAATTCCTCTTGACATT
401      H  E  N  K  L  L  Q  R  M  A  I  S  I  E  R  L  S  S  K  V  R  E  C  F  L  D  L  G  C  F  P  E  D  K  R  I  P  L  D  I

1321    CTCATCAATGTTTGGAAGGAGTTACATGATCTTGATGACGAAGAAGCTCTTGCTGTTCTTTTCGAGTTATCTCAGAAGAATCTTCTTACGTTGGTCAAAGATGCACGCGGTGGTGACATT
441      L  I  N  V  W  K  E  L  H  D  L  D  D  E  E  A  L  A  V  L  F  E  L  S  Q  K  N  L  L  T  L  V  K  D  A  R  G  G  D  I

1441    TATAGCAGTTATTATGAGATGTATGTCACTCAACACGATGTATTAAGGGACCTTGCCCTTCATTTCAGTTGCCAGGAGAATGTGAACGACCGCAAGCGATTACTGATGCCAAAAAGCGAC
481      Y  S  S  Y  Y  E  M  Y  V  T  Q  H  D  V  L  R  D  L  A  L  H  F  S  C  Q  E  N  V  N  D  R  K  R  L  L  M  P  K  S  D

1561    ACAGAGCTTCCAAAAGAATGGTTAAGGAAATCGGAACAGCCATTTAATGCCCAACTTGTTTCAATTCACACAGGTGAAATGGAAGAAATGGATTGGGCGCCTATGATATTTCCTGAAGCT
521      T  E  L  P  K  E  W  L  R  K  S  E  Q  P  F  N  A  Q  L  V  S  I  H  T  G  E  M  E  E  M  D  W  A  P  M  I  F  P  E  A

1681    AAAGTGCTCATTTTAAACTTCTCCTCGAGTGGATACTTCTTGCCTTCTTTTCTTTGCAACATGCCGAAGATAAGAGCATTAATTGTGCTAAATAACAATGCAACACATGCAACTCTCACC
561      K  V  L  I  L  N  F  S  S  S  G  Y  F  L  P  S  F  L  C  N  M  P  K  I  R  A  L  I  V  L  N  N  N  A  T  H  A  T  L  T

1801    AATTTCTCAGTTTTTTCTAGTTTGGTCAACTTGAGAGGCATCTGGCTGGAAAAAATTTCCATGACACAACTATTCGATGCTTGCACGCCATTGAAACATCTAAGGAAGCTATCTCTTGTT
601      N  F  S  V  F  S  S  L  V  N  L  R  G  I  W  L  E  K  I  S  M  T  Q  L  F  D  A  C  T  P  L  K  H  L  R  K  L  S  L  V

1921    TTCTGCAAGATCAACAACAGCCTCGACGAGTGGGCGGTAGATGTATCCCAGATCTTCCCGTTTCTTTTCGAACTCAAAATTGATCACTGCAACGACTTGCGTAAGCTACCTTCAAGCATT
641      F  C  K  I  N  N  S  L  D  E  W  A  V  D  V  S  Q  I  F  P  F  L  F  E  L  K  I  D  H  C  N  D  L  R  K  L  P  S  S  I

2041    TGTGAGATGCAAAGTCTCAAGTGTCTTAGTGTCACCAACTGTCATAATCTCAGTCAACTCCCTACCAACTTATGGAAGCTGAAAAATCTACAAATCTTGAGACTTTTTGCTTGCCCACTC
681      C  E  M  Q  S  L  K  C  L  S  V  T  N  C  H  N  L  S  Q  L  P  T  N  L  W  K  L  K  N  L  Q  I  L  R  L  F  A  C  P  L

2161    CTCAAAACTCTATCCCCAAGCATTTGTGTACTTTCTTGTCTAAAGTACATTGACATCTCCCAATGTGTTTACTTAACCAGCCTTCCTGAAGAAATTGGCAAGCTGACAAGCCTAGAGAAA
721      L  K  T  L  S  P  S  I  C  V  L  S  C  L  K  Y  I  D  I  S  Q  C  V  Y  L  T  S  L  P  E  E  I  G  K  L  T  S  L  E  K

2281    ATTGACATGAGAGAATGCTCACTCATAAGGAGACTACCTAGATCAGTTGTGTCTTTGCAATCTCTCTGTCACGTAATCTGCGAAGAAGACGTCTCGTGGCTATGGGAGGATTTGAAGAGT
761      I  D  M  R  E  C  S  L  I  R  R  L  P  R  S  V  V  S  L  Q  S  L  C  H  V  I  C  E  E  D  V  S  W  L  W  E  D  L  K  S

2401    CATATGCCTAATTTGTACATTCAAGTCGCCGAGAAATGCTTCAACTTAGATTGGCTCAAAGAGTGA
801      H  M  P  N  L  Y  I  Q  V  A  E  K  C  F  N  L  D  W  L  K  E  *
```

Figure S2. Nucleotide and deduced amino acid sequences of cucumber *CsRSF2* CDS encoding a protein. The deduced amino acid sequences are given below the nucleotide sequences. The transcriptional start site is shown in red and the termination codon is marked by an asterisk (*).

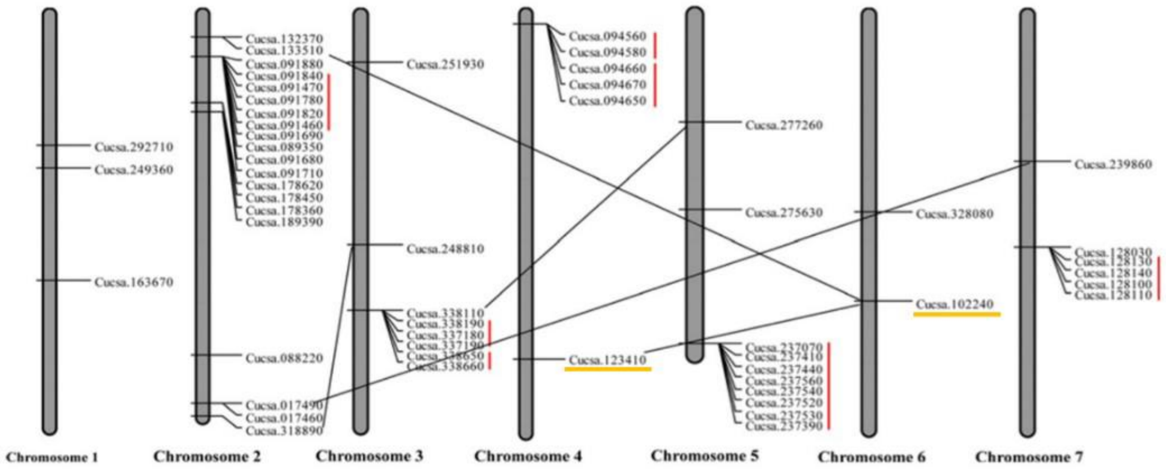

Figure S3. Chromosome location of NBS-LRR superfamily in the cucumber. The yellow lines represent *CsRSF1* (Cucsa.102240) and *CsRSF2* (Cucsa.123410).

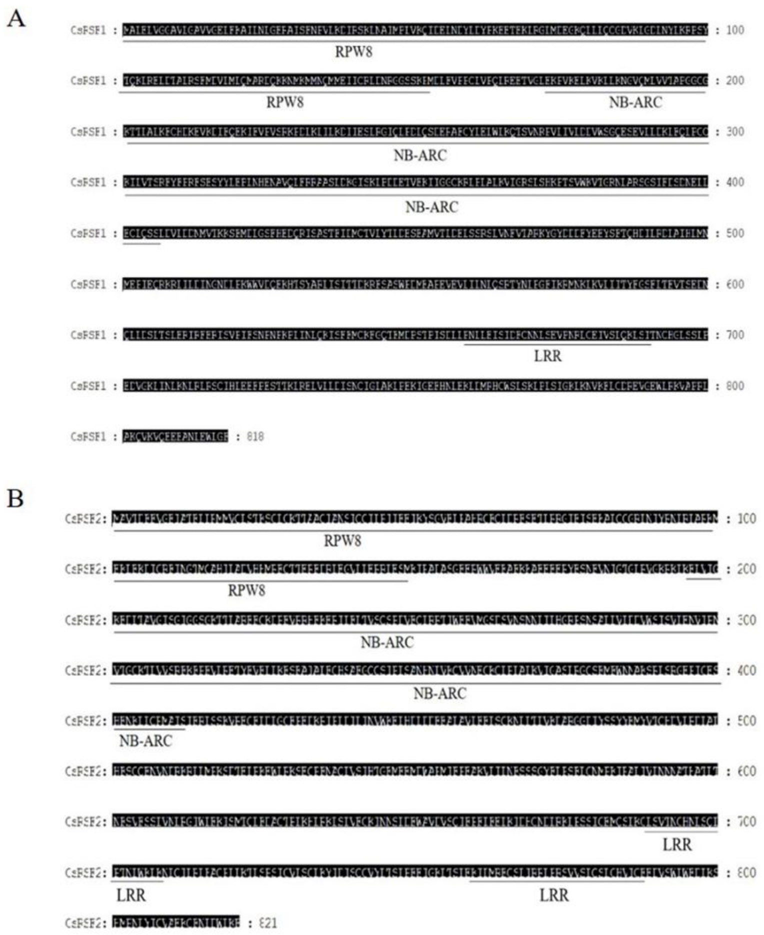

Figure S4. The structural domain of CsRSF protein. (A) The structural domain of CsRSF1 protein. (B) The structural domain of CsRSF2 protein. Lines represent the sequences of NB-ARC (NBS), LRR and RPW8 domains.

Supplementary Tables

Table S1 The primers of PCR used in this study.

Analysis	Primer name	Sequence(5'-3')
PCR for *CsRSF1*	*CsRSF1*-F<br>*CsRSF1*-R	TTGATACATATGCCCGTCGACATGGCGCTGGAATTGGTG<br>GCCCTTGCTCACCATGGATCCAAAACCAAGCCACTCCAGGTT
PCR for *CsRSF2*	*CsRSF2*-F<br>*CsRSF2*-R	TTGATACATATGCCCGTCGACATGGCGGTTACAGATTTCTTTGT<br>GCCCTTGCTCACCATGGATCCCTCTTTGAGCCAATCTAAGTTGAAG
CsRSF1-silencing vector	TRV*CsRSF1*-F<br>TRV*CsRSF1*-R	AAGGTTACCGAATTCTCTAGAGCTTCTTCCTCTTGCACTTTCA<br>GGGACATGCCCGGGCGCTCGAGGTCCTGCTTGATATATCTAACTG
CsRSF2-silencing vector	TRV*CsRSF2*-F<br>TRV*CsRSF2*-R	AAGGTTACCGAATTCTCTAGATCACTCTTTGAGCCAATCTAAG<br>GGGACATGCCCGGGCGCTCGAGAAGCATTTGTGTACTTTCTTGT
CsRSF1-overexpression vector	GFP*CsRSF1*-F<br>GFP*CsRSF1*-R	TTGATACATATGCCCGTCGACATGGCGCTGGAATTGGTG<br>GCCCTTGCTCACCATGGATCCAAAACCAAGCCACTCCAGGTT
CsRSF2-overexpression vector	GFP*CsRSF2*-F<br>GFP*CsRSF2*-R	TTGATACATATGCCCGTCGACATGGCGGTTACAGATTTCTTTGT<br>GCCCTTGCTCACCATGGATCCCTCTTTGAGCCAATCTAAGTTGAAG
Chimeric primer for *CsRSF1*-GFP	*CsRSF1*-GFP-F<br>*CsRSF1*-GFP-R	CAAGCTGCCACTGTCGATTG<br>TGGTGCAGATGAACTTCAGGGT
Chimeric primer for *CsRSF2*-GFP	*CsRSF2*-GFP-F<br>*CsRSF2*-GFP-R	TACCTAGATCAGTTGTGTCTTTGCA<br>TGGTGCAGATGAACTTCAGGGT

Table S2 The primers of qRT-PCR used in this study.

Analysis	Primer name	Sequence(5'-3')
CsActin gene for qRT-PCR	Actin-F<br>Actin-R	TCGTGCTGGATTCTGGTG<br>GGCAGTGGTGGTGAACAT
qRT-PCR for *CsRSF1*	q-*CsRSF1*-F<br>q-*CsRSF1*-R	CTACCTTCATTGACATGTGCAC<br>TTCGAGAGGATAGTTCGTCAAG
qRT-PCR for *CsRSF2*	q-*CsRSF2*-F<br>q-*CsRSF2*-R	CGGGGAAGACTACTTTAGCTAG<br>GTTTTGCAACCAGTTACGTTTG
Chitinase for qRT-RCR in transgenic cucumber	*Chitinase*-F<br>*Chitinase*-R	GCCGCAGTGTCCAATACCAG<br>TCAGGAGATTGTCCGCGTTA
CuPi1 for qRT-RCR in transgenic cucumber	*CuPi1*-F<br>*CuPi1*-R	GCACCAAAACAACGAAAAGG<br>GGCTATAAGGACCGCTACCAT
PR-1a for qRT-RCR in transgenic cucumber	*PR-1a*-F<br>*PR-1a*-R	GAACTCTGGCGGACCTTA<br>GCATCTCACTTTGGCACATC
